# Metastasis and basement membrane-related signature enhances hepatocellular carcinoma prognosis and diagnosis by integrating single-cell RNA sequencing analysis and immune microenvironment assessment

**DOI:** 10.1186/s12967-024-05493-0

**Published:** 2024-07-31

**Authors:** Shijia Wei, Jingyi Tan, Xueshan Huang, Kai Zhuang, Weijian Qiu, Mei Chen, Xiaoxia Ye, Minhua Wu

**Affiliations:** 1 The First Clinical Medical College, Guangdong Medical University, Zhanjiang, 524000 China; 2 School of Pharmacy, Guangdong Medical University, Zhanjiang, 524000 China; 3 School of Basic Medicine, Guangdong Medical University, Zhanjiang, 524000 China; 4 School of Public Health, Guangdong Medical University, Dongguan, 523808 China

**Keywords:** Hepatocellular carcinoma, Metastasis, Basement membrane, Prognostic model, Immunotherapy Response, ScRNA-seq

## Abstract

**Background:**

Hepatocellular carcinoma (HCC) is the most common type of primary liver cancer and second leading cause of cancer-related deaths worldwide. The heightened mortality associated with HCC is largely attributed to its propensity for metastasis, which cannot be achieved without remodeling or loss of the basement membrane (BM). Despite advancements in targeted therapies and immunotherapies, resistance and limited efficacy in late-stage HCC underscore the urgent need for better therapeutic options and early diagnostic biomarkers. Our study aimed to address these gaps by investigating and evaluating potential biomarkers to improve survival outcomes and treatment efficacy in patients with HCC.

**Method:**

In this study, we collected the transcriptome sequencing, clinical, and mutation data of 424 patients with HCC from The Cancer Genome Atlas (TCGA) and 240 from the International Cancer Genome Consortium (ICGC) databases. We then constructed and validated a prognostic model based on metastasis and basement membrane-related genes (MBRGs) using univariate and multivariate Cox regression analyses. Five immune-related algorithms (CIBERSORT, QUANTISEQ, MCP counter, ssGSEA, and TIMER) were then utilized to examine the immune landscape and activity across high- and low-risk groups. We also analyzed Tumor Mutation Burden (TMB) values, Tumor Immune Dysfunction and Exclusion (TIDE) scores, mutation frequency, and immune checkpoint gene expression to evaluate immune treatment sensitivity. We analyzed integrin subunit alpha 3 (*ITGA3*) expression in HCC by performing single-cell RNA sequencing (scRNA-seq) analysis using the TISCH 2.0 database. Lastly, wound healing and transwell assays were conducted to elucidate the role of *ITGA3* in tumor metastasis.

**Results:**

Patients with HCC were categorized into high- and low-risk groups based on the median values, with higher risk scores indicating worse overall survival. Five immune-related algorithms revealed that the abundance of immune cells, particularly T cells, was greater in the high-risk group than in the low-risk group. The high-risk group also exhibited a higher TMB value, mutation frequency, and immune checkpoint gene expression and a lower tumor TIDE score, suggesting the potential for better immunotherapy outcomes. Additionally, scRNA-seq analysis revealed higher *ITGA3* expression in tumor cells compared with normal hepatocytes. Wound healing scratch and transwell cell migration assays revealed that overexpression of the MBRG *ITGA3* enhanced migration of HCC HepG2 cells.

**Conclusion:**

This study established a direct molecular correlation between metastasis and BM, encompassing clinical features, tumor microenvironment, and immune response, thereby offering valuable insights for predicting clinical outcomes and immunotherapy responses in HCC.

**Supplementary Information:**

The online version contains supplementary material available at 10.1186/s12967-024-05493-0.

## Background

Hepatocellular carcinoma (HCC) is the most common primary liver cancer and the second leading cause of cancer-related deaths worldwide [1]. Due to its rapid growth and early metastasis, the mortality rate for HCC is extremely high, especially in patients with tumor metastasis [2]. Early detection and diagnosis play a pivotal role in determining the prognosis of patients with cancer, and early treatments such as surgical resection and liver transplantation offer a potential cure yet remain challenging due to the asymptomatic nature of early-stage HCC. In recent years, progress has been made in the treatment of HCC through targeted therapies such as sorafenib and lenvatinib, which target tumor growth pathways, and immune checkpoint inhibitors (ICIs) like atilizumab and bevacizumab, which, compared to classic first-line sorafenib-based chemotherapy, are associated with a better prognosis [3, 4]. However, despite these advancements, resistance to treatments like sorafenib and limited efficacy in late-stage HCC highlight the urgent need for improved therapeutic options and biomarkers for the monitoring and early diagnosis of HCC, which have not exhibited significant improvements [5]. Therefore, this study aimed to investigate and evaluate potential biomarkers to provide robust support for improving the survival outcomes and treatment efficacy in patients with HCC, with a particular focus on overcoming drug resistance, enhancing early detection, and offering individualized treatment plans.

Tumor metastasis manifests as the penetration of tumor cells through the basement membrane (BM) from the primary lesion site to distant organs to form new metastases [6]. The BM is a thin layer of extracellular matrix located beneath epithelial and endothelial tissues. It serves as a structural barrier, primarily composed of laminin and collagen IV proteins, which hinder the invasion, intravasation, and extravasation of cancer cells [7]. The BM is also considered a major obstacle that tumor cells must overcome repeatedly to facilitate the completion of metastasis [7]. Recent studies have shown that tumor cells can invade by altering the stiffness and tension of the BM [8]. In addition, upregulation of *COL4A1*, a gene encoding collagen IV, promotes the proliferation and metastasis of HCC cells via FAK-Src signaling [9], and overexpression of *LAMC1* and *LAMA4*, genes encoding subunits of laminin, predicts poor prognosis and enhances HCC cell invasion and migration [10, 11]. Notably, some studies suggest that the expression of the BM genes *CTSA*, *ITGA6*, *ITGA8*, and *LAMC1* in HCC is significantly elevated compared to that in normal tissues, both at the mRNA and protein levels, and these genes therefore have an extremely high diagnostic value [12]. Moreover, the expression of the metastasis genes *SIX4*, *PRMT9*, and *ONECUT2* are important risk factors for recurrence and survival in patients with HCC and could serve as prognostic biomarkers [13–15]. Although both metastasis and BM genes expression have significant prognostic significance in HCC, prognostic models that combine both metastasis and BM genes to elucidate the overall impact of these factors on the prognostic significance, characteristics of the tumor microenvironment (TME), immunotherapy, and metastasis of HCC are lacking.

TME is closely associated with tumorigenesis and the escape of tumor cells from the immune system, as well as the efficacy and clinical prognosis of tumors [16]. The mechanism of tumor metastasis involving the BM has been shown to be closely related to the TME. Previous literature has indicated that the expression of BM genes is positively correlated with immune scores in HCC, suggesting that BM genes have a similar impact in the TME [16]. For example, in addition to secreting signaling molecules that facilitate tumor cell invasion, immune cells can traverse the BM. Additionally, numerous studies have demonstrated that T cells can be stimulated, proliferate, adhere, and migrate under the regulation of various components of the BM, thereby promoting tumor growth and metastasis [17–20]. In particular, regulatory T cells (Tregs) indirectly promote tumor cell metastasis by suppressing antitumor immune responses [18]. Furthermore, macrophages promote cancer cell endocytosis [19] and degrade the BM by secreting several matrix metalloproteinases (MMPs) [20]. Notably, in HCC, cohorts with high expression of the BM gene MMP9 are more susceptible to immunotherapy and exhibit increased CD8 T-cell infiltration with functional failure due to high expression of immune checkpoints. Thus, MMP9 may serve as a predictor of the immune profile of HCC and the response to immunotherapy [21]. Although significant research has investigated the impact of the TME on cancer, whether immune cells can enhance the invasion of HCC by mechanically modifying the BM is still not fully understood. Therefore, assessing the immunological status of HCC based on metastasis and BM genes is imperative to advance the development of immunotherapies and enhance the prognosis of patients with cancer.

In our study, a prognostic model consisting of 12 genes was constructed for HCC based on metastasis and basement membrane**-**related genes (MBRGs), one of which, *ITGA3*, was further analyzed to investigate its novel role in the invasion of human HCC HepG2cells. Physicians should use the results of this study to provide more precise and individualized therapeutic choices for patients with HCC.

## Materials and methods

### Data resources

A total of 424 HCC patients were screened from The Cancer Genome Atlas (TCGA) database (https://portal.gdc.cancer.gov/). The liver hepatocellular carcinoma (LIHC)-Japan (JP) cohort, obtained from the International Cancer Genome Consortium (ICGC) database (https://dcc.icgc.org/), was used for external validation. Expression data, clinical information, and mutation data of the 424 HCC samples(374 tumor samples and 50 normal samples) for signature establishment were obtained from TCGA. The expression data and clinical information of 240 HCC samples for external validation were obtained from the LIHC-JP cohort acquired from the ICGC database. The BM genes were obtained from previous research [22]. Metastasis genes were obtained from the Human Cancer Metastasis Database (HCMDB; https://ngdc.cncb.ac.cn/) [23].

### Differential analysis and univariate cox regression analysis in TCGA-LIHC

We initially obtained 424 TCGA-LIHC patient samples (374 tumor and 50 normal patients) containing 59,427 genes (FPKM ) from the TCGA database. Subsequently, we performed differential analysis of these genes utilizing the “limma” R package [24]. Applying the following screening criteria for differential analysis of variance: log2|Fold change| ≥ 1 and false discovery rate (FDR) < 0.05. Ultimately, we obtained 13,095 differentially expressed genes (DEGs) in TCGA-LIHC. We obtained 2165 metastasis genes from the HCMDB database and 224 BM genes from previous research [22]. We took the intersection of the 13,095 DEGs, 2165 metastasis genes, and 224 BM genes from TCGA-LIHC and concluded with 35 differentially expressed metastasis and BM genes. Next, 12 DEGs related to prognosis in metastasis and BM were obtained utilizing univariate Cox regression analysis (*p* < 0.05) [25].

### Construction and validation of a prognostic model for MBRGs

We further constructed a prognostic risk model on the basis of 12 MBRGs using multivariate Cox regression analysis with the 12 prognostic-related metastasis and BM genes derived from the univariate Cox regression analysis [26]. The risk score was determined using standardized HCC mRNA expression data from TCGA dataset and was calculated using the following equation: patient risk score =∑(each gene expression level × corresponding coefficient) [27]. Patients with HCC were divided into high- and low-risk groups according to the median risk score, and their overall survival (OS) was compared [28]. The LIHC cohort from the ICGC database was used for validation. The expression levels of each MBRG were normalized, and the risk score was calculated using the aforementioned formula. Subsequently, patients with LIHC in the ICGC cohort were divided into high- and low-risk groups based on median risk scores, allowing for the comparison of OS between the two groups. Additionally, the “stats” R package was employed to conduct principal component analysis (PCA) and t-distributed stochastic neighbor embedding (t-SNE) on the MBRGs prognostic model for patients in the high- and low-risk groups, as previously described [29].

### Nomogram construction and independent prognostic analysis of TCGA-LIHC data

Univariate and multivariate Cox regression analyses were used to identify independent risk factors in patients with HCC. Based on the results of the multivariate Cox analyses, the R package “RMS” was applied to create nomogram to guide clinical decision-making. We constructed a nomogram using age, grade, and tumor-node-metastasis (TNM) staging combined with risk scores in TCGA-LIHC dataset and further validated its accuracy using 1-, 2- and 3-year calibration curves [30]. Finally, the concordance index calibration method was used to verify the accuracy of our prognostic model.

### Functional enrichment and gene set enrichment analyses

Gene Ontology (GO) and Kyoto Encyclopedia of Genes Genomes (KEGG) analyses were performed on the high- and low-risk groups of patients with HCC using the “clusterProfiler” R package [31]. Subsequently, gene set enrichment analysis (GSEA) was conducted to identify the gene sets (c2.cp.kegg.v2022.1.Hs.symbols.gmt) (MSigDB database, https://www.gsea-msigdb.org) with consistent but insignificant differential expression trends to explore the relationship between the epithelial-mesenchymal transition (EMT) pathway which involves cells penetrating the BM and confers metastatic properties upon cancer cells, and the expression levels of each prognostic gene [32].

### Immunological analysis and immunotherapy

The single-sample GSEA (ssGSEA) algorithm was used to analyze the abundance of 16 immune cell types in each LIHC sample, implemented through the “gsva” R package. The immune landscape of the high- and low-risk groups of the MBRGs model was evaluated using the Cell-type Identification By Estimating Relative Subsets Of RNA Transcripts (CIBERSORT) [33], Quantifying Tumor Immune Signature Events (QUANTISEQ) [34], Microenvironment Cell Populations (MCP) counter [35], ssGSEA [36], and Tumor Immune Estimation Resource (TIMER) [37] algorithms. The expression of immune checkpoint genes was examined for differences between the two groups to evaluate treatment sensitivity.

### Mutation analysis

The mutation data of patients from the TCGA cohort were extracted for the analysis of mutation conditions and gene copy number variations (CNV) of prognostic genes. Survival was estimated for patients in both the high- and low-risk groups, as well as for those with high and low mutation burdens. Somatic mutations in TCGA high- and low-risk groups were analyzed using the “mafTools” package in R [38]. Subsequently, the tumor mutation burden (TMB) of the two groups of patients was evaluated.

### Drug sensitivity analyses

The drug half-maximal inhibitor concentration (IC50) analysis for the high- and low-risk groups of patients with HCC was conducted using the “pRRophetic” package in R [39], based on the Genomics of Drug Sensitivity in Cancer database (GDSC, https://www.cancerrxgene.org/) [40], and we analyzed the gene expression of specific drug targets using the DrugBank database (www.drugbank.ca).

### Protein-protein interaction network

A protein-protein interaction (PPI) network was developed using the GeneMAINA database (https://genemania.org/) to analyze the co-expression of and interaction between key proteins.

### Single cell RNA sequencing analysis based on the tumor immune single-cell hub 2.0 database

We utilized the Tumor Immune Single-cell Hub 2.0 (TISCH 2.0) database (http://tisch.comp-genomics.org/) for single-cell RNA sequencing (scRNA-seq) analysis of *ITGA3* in HCC [41]. We acquired two HCC datasets from this database, GSE166635 [42], which contains 22,631 cells from two samples, and GSE146409 [43] which contains 2916 cells from six samples.

### Immunohistochemical analysis

The Human Protein Atlas (HPA) database (https://www.proteinatlas.org/) is a comprehensive proteome atlas that provides information on the distribution of proteins in human tissues and cells. We downloaded immunohistochemical images of tumor tissues and their corresponding normal tissues from the HPA to analyze the differential expression of *ITGA3* at the protein level [44].

### Cell lines and culture

The human HCC cell line HepG2 was purchased from Procell Biotech (Wuhan, China). The cells were cultured in Dulbecco’s Modified Eagle Medium (DMEM) high-sugar medium (Procell, Wuhan, China) supplemented with 10% fetal bovine serum (Procell, Wuhan, China) at 37 °C in a 5% CO_2_ atmosphere.

### Lentiviral vector transfection

*ITGA3*-overxpressing (*ITGA3*-OE) and non-targeted control (vector) lentiviral vectors were constructed and packaged by LeapWal Biotech (Hunan, China). HepG2 cells at 50% confluency were transfected with lentiviral particles using polybrene according to the manufacturer’s guidelines.

### Reverse transcription-quantitative PCR

After incubation with *ITGA3*-OE or vector lentiviral particles for 16 h, the RNA isolation reagent TRIzol (Takara, Dalian, China) was used to extract total RNA from the cells for reverse transcription-quantitative PCR (RT-qPCR). cDNA was obtained from mRNA with a Primescript™ RT reagent kit (Takara). A SYBR Green PCR kit (GeneCoepia) was used for amplification using the Light Cycler systemABI QuantStudio1 (Thermo Fisher Scientific, USA). The primers used for amplification of *ITGA3* and the internal control *β*-*actin* were as follows: sense 5′-CTACGAAGTCAGCAATGGCAAGTG-3′ and antisense 5′-GGTTGATAAGGTCTCCAGGTGGTC-3′ for *ITGA3*; sense 5′-TGACGTGGACATCCGCAAAG-3′ and antisense 5′-CTGGAAGGTGGACAGCGAGG-3′ for *β-actin*. The cycling program used was as follows: denaturation at 95 °C for 30 s, followed by 40 cycles of denaturing at 95 °C for 10 s, and annealing at 60 °C for 30 s. The results were analyzed using the 2^−ΔΔCt^ method [45].

### Cell proliferation assay

Approximately 5 × 10^3^ HepG2 cells were seeded in 6-well plates and incubated with *ITGA3*-OE or vector lentiviral particles. After incubation for 24 and 48 h, cell counting kit-8 (CCK-8) reagent (Beyotime, Shanghai, China) was added to each well, and then the cells were incubated for another 2 h at 37 °C. The optical density value of each well was measured at 450 nm using a microplate reader (BioTek, Winooski, VT, USA) [46].

### Wound healing scratch assay

After incubation with *ITGA3*-OE or vector lentiviral particles in a 6-well plate, HepG2 cells were serum-starved and artificial scratch wounds were made using a 200-µL pipette tip. The cells were then washed twice with phosphate-buffered saline. Cell migration was imaged at the indicated time points.

### Transwell cell migration assay

HepG2 cells (1 × 10^5^) transfected with *ITGA3*-OE or vector lentiviral particles for 16 h were suspended in 200 µL of serum-free DMEM high sugar medium and then added to the upper chamber of a transwell migration plate, and 600 µL of complete media was added to the lower chamber. After 48 h of incubation, the cells in the upper chamber were removed using cotton swabs, and the cells that migrated through the polyvinylidene fluoride membrane were fixed in 4% paraformaldehyde for 10 min, stained with crystal violet dye (Solarbio, Beijing, China), and counted under a microscope (three fields per chamber).

### Data analysis

All statistical analyses were conducted using R software (version 4.2). Gene expression levels between tumor and adjacent normal tissues were compared using independent-sample t-tests. Specifically, we examined the differences in immune cell infiltration and activation of immune pathways between these two groups, and the statistical significance of the proportional differences was assessed using chi-squared tests. The coefficients of the prognostic characteristics were calculated using multivariable Cox regression analysis. Kaplan-Meier curves were used to generate survival curves for the high- and low-risk groups, and we used Pearson’s correlation test to analyze the correlation between variables, employing the log-rank test to assess the statistical significance of the differences. Univariate and multivariate Cox regression analyses were conducted to determine the independent prognostic factors for OS, with variables reaching statistical significance used in the multivariate Cox proportional hazards model. Statistical significance was set at *p* < 0.05 for all tests. Our flowchart was created using Biorender website (biorender.com). Clinical features charts were created using Xiantao bioinformatics tool (http://www.xiantaozi.com/).

## Results

### Identification and analysis of prognostic genes

**Fig. 1:**
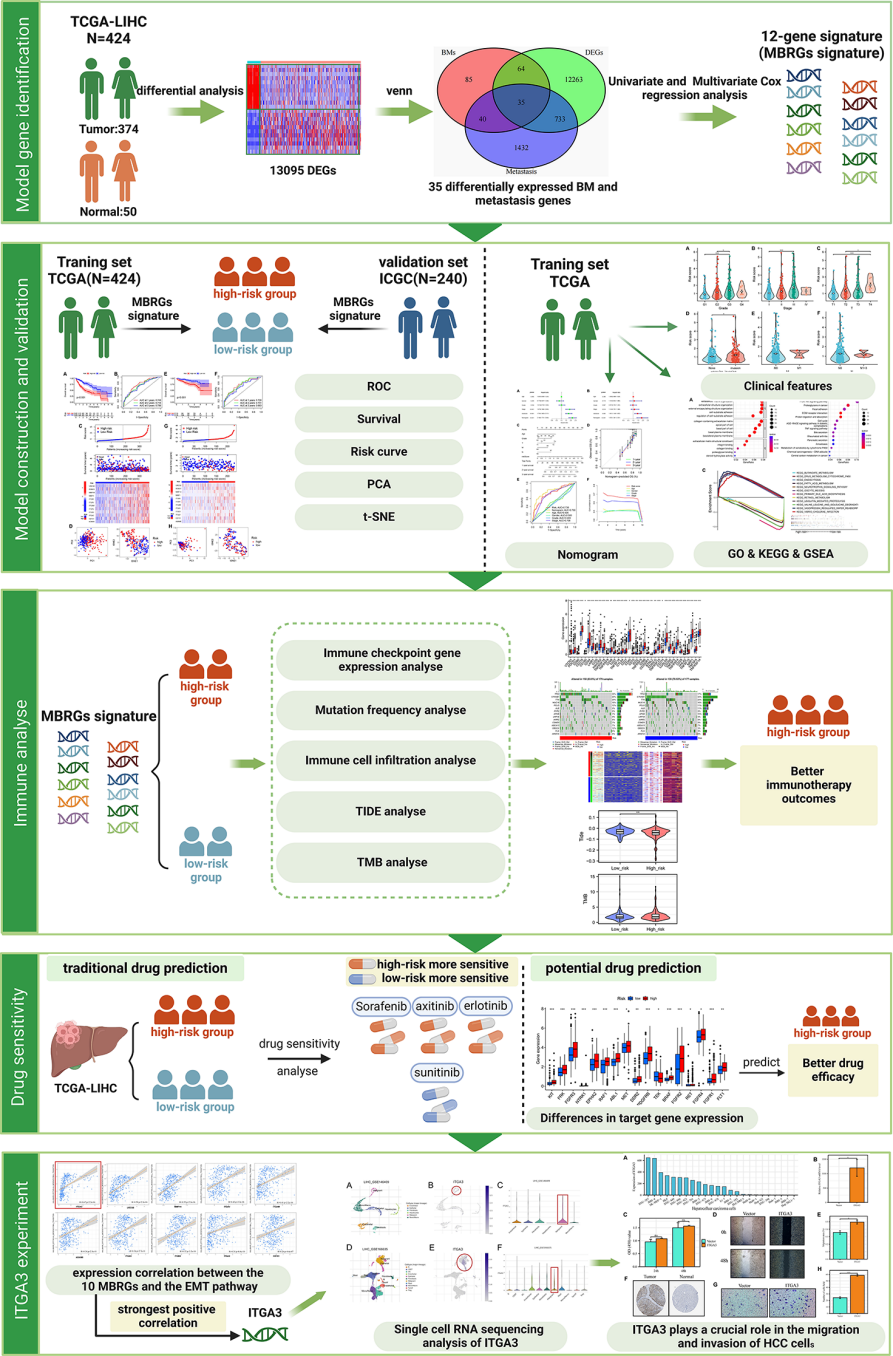
Workflow chart of the study, created with BioRender. Abbreviations: BM, basement membrane; CNV, copy number variable; GO, Gene Ontology; EMT, epithelial-mesenchymal transition; GSEA, gene set enrichment analysis; ICGC, International Cancer Genome Consortium; ITGA3, integrin subunit alpha 3; KEGG, Kyoto Encyclopedia of Genes and Genomes; MBRGs, metastasis and basement membrane-related genes; PCA, principal component analysis; ROC, receiver operating characteristic; ssGSEA, single-sample gene set enrichment analysis; TCGA, The Cancer Genome Atlas; TIDE, tumor immune dysfunction and exclusion; TMB, tumor mutation burden; t-SNE, t-distributed stochastic neighbor embedding

**Fig. 2:**
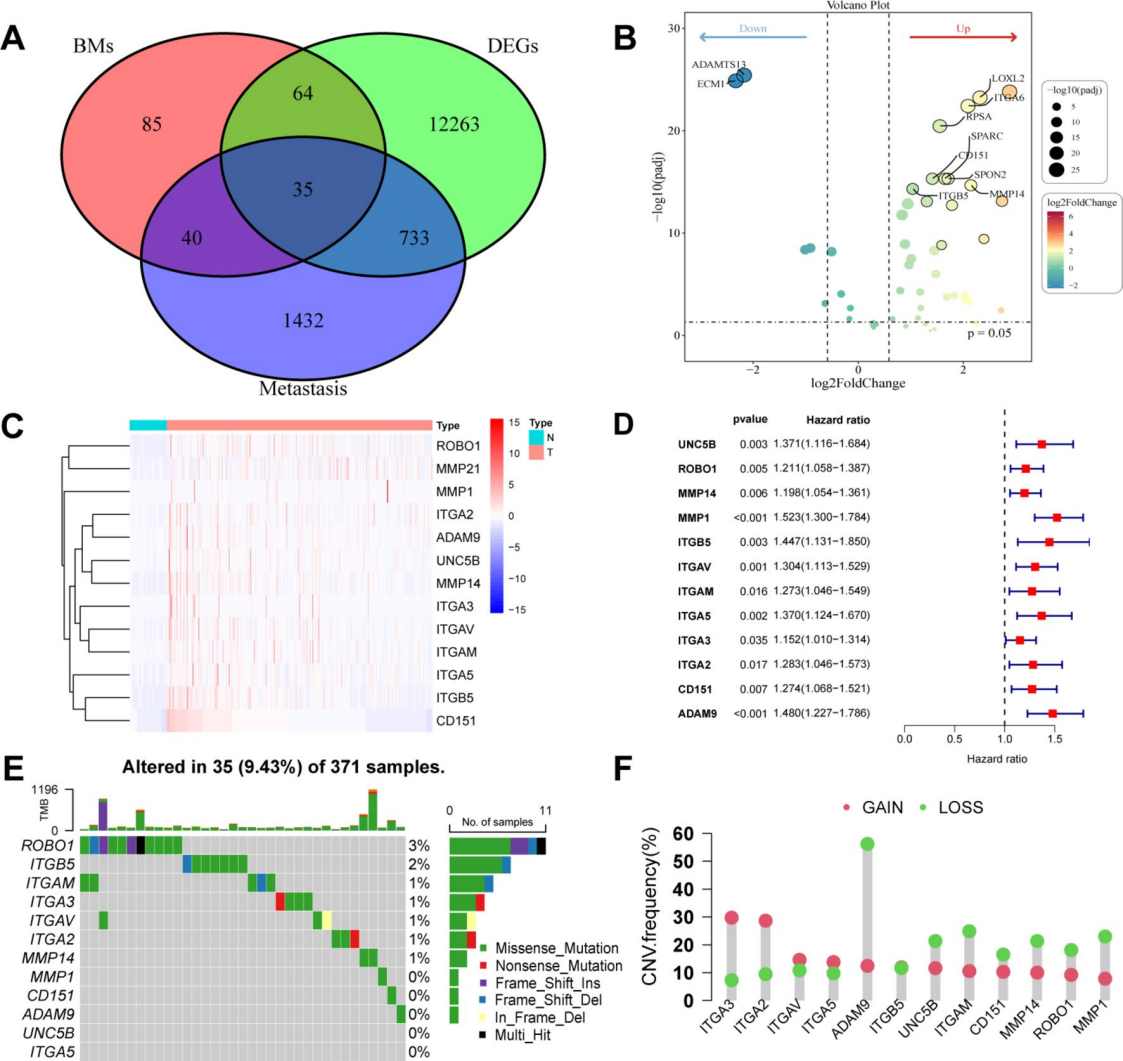
Prognosis-related DEGs screened in TCGA-LIHC cohort. **(A)** Venn diagram illustrating the overlap of datasets. **(B)** Volcano plot of DEGs related to prognosis in metastasis and BM between tumor and normal tissues. **(C)** Comparative expression levels of 12 DEGs related to prognosis in metastasis and BM in tumor tissues versus normal tissues. **(D)** Forest plot demonstrating the prognostic value of 12 DEGs related to prognosis in metastasis and BM for HCC. **(E)** MAF tool analysis of 12 DEGs related to prognosis in metastasis and BM. **(F)** CNV analysis of 12 DEGs related to prognosis in metastasis and BM. Abbreviations: BM, basement membrane; CNV, copy number variable; DEG, differentially expressed gene; HCC, hepatocellular carcinoma; LIHC, liver hepatocellular carcinoma; MAF, mutation annotation format; TCGA, The Cancer Genome Atlas

Figure 1 shows the workflow chart of the study. We initially obtained 424 TCGA-LIHC patient samples (374 tumor and 50 normal) containing 59,427 genes (FPKM) from the TCGA database. Subsequently, we performed differential analysis of these genes, applying the screening criteria of log2 |Fold change| ≥ 1 and FDR < 0.05 for differential analysis of variance. Ultimately, we obtained 13,095 DEGs in TCGA-LIHC. We obtained 2165 metastasis genes from the HCMDB database and 224 BM genes from previous research. We took the intersection of the 13,095 DEGs, 2165 metastasis genes, and 224 BM genes from TCGA-LIHC and concluded with 35 differentially expressed metastasis and BM intersection genes (Fig. 2A). Among the 35 genes, 32 were up-regulated and 3 were down-regulated (Fig. 2B). Subsequently, univariate Cox regression analysis of these 35 differentially expressed metastasis and BM intersection genes was used to identify 12 prognostic genes, all of which were significantly related to OS, with hazard ratio > 1 in HCC (all *p* < 0.05). In addition, 12 prognostic genes were upregulated in tumor tissues (Fig. 2C, D). The correlation network results showed a positive correlation between the prognostic genes (Fig. S1). These results suggest that the high expression of prognostic genes predict a poor prognosis. Next, the mutation status of prognostic genes and the incidence of CNVs in HCC were assessed; out of 371 HCC cases, 35 (9.43%) had mutations in metastasis- and BM-related genes, including *ROBO1* (3%), *ITGB5* (2%), *ITGAM* (1%), *ITGA3* (1%), *ITGAV* (1%), *ITGA2* (1%), and *MMP14* (1%) (Fig. 2E). In addition, CNVs were prevalent in most of the prognostic genes. Among them, *ITGA3* and *ITGA2* were mainly subjected to amplifications, whereas *ADAM9*, *ITGAM*, *MMP1* and *MMP14* were mainly subjected to deletions (Fig. 2F). Subsequently, we speculated on the survival analyses of the prognostic genes and found that the expression levels of prognostic genes were significantly different for patient survival, with elevated expression of the prognostic genes indicating a poorer prognosis (Fig. S2) (all *p* < 0.01).

### Construction of an MBRGs prognostic model in TCGA database

Using the 12 prognostic genes derived from univariate Cox regression analysis, we further developed a prognostic model based on these 12 MRBGs using multivariate Cox regression analysis. Multivariate Cox analysis was performed to obtain the risk coefficients of each MBRG, which are shown in Table S1. The risk score was calculated using the following formula, wherein “exp.” denotes the expression level of the corresponding MBRG:

$$\eqalign{{\rm{Riskcore}}& = {\rm{(0}}{\rm{.556*UNC5B\; exp}}{\rm{.)}} + {\rm{ (0}}{\rm{.1291* ROBO1\; exp}}{\rm{.)}} \cr &+ {\rm{(0}}{\rm{.1482* MMP14\; exp}}{\rm{.)}} + {\rm{ (0}}{\rm{.7548*MMP1\; exp}}{\rm{.)}} \cr &+ {\rm{ (0}}{\rm{.3003* ITGB5\; exp}}{\rm{.)}} + {\rm{(0}}{\rm{.4213*ITGAV\; exp}}{\rm{.)}} \cr &+ {\rm{(}} - {\rm{0}}{\rm{.2984*ITGAM\; exp}}{\rm{.)}} + {\rm{(}} - {\rm{0}}{\rm{.3605*ITGA5\; exp}}{\rm{.)}} \cr &+ {\rm{(}} - {\rm{0}}{\rm{.7239*ITGA3\; exp}}{\rm{.)}} + {\rm{(}} - {\rm{0}}{\rm{.1902*ITGA2\; exp}}{\rm{.)}} \cr &+ {\rm{(0}}{\rm{.9724*CD151\; exp}}{\rm{.) + (0}}{\rm{.6254*ADAM9\; exp}}{\rm{.)}} \cr}$$

**Fig. 3:**
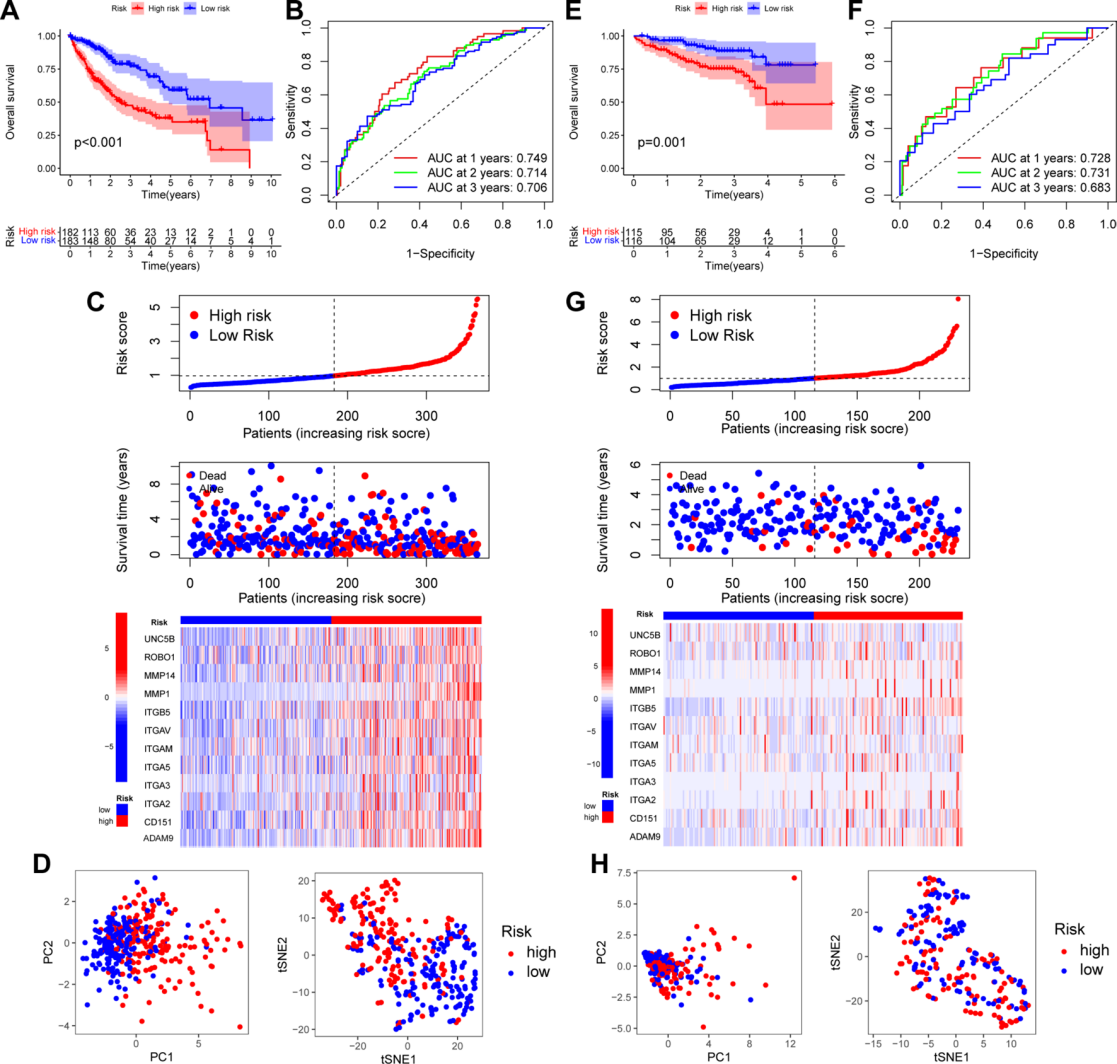
Construction and validation of the MBRGs prognostic model. **(A)** Kaplan-Meier curve displaying OS of TCGA dataset. **(B)** ROC curve for TCGA dataset. **(C)** Risk curve and survival status for TCGA dataset. **(D)** PCA and t-SNE graph for TCGA dataset. **(E)** Kaplan-Meier curve displaying OS of the ICGC dataset. **(F)** ROC curve for the ICGC dataset. **(G)** Risk curve and survival status for the ICGC dataset. **(H)** PCA and t-SNE graph for the ICGC dataset. Abbreviations: AUC, area under the curve; ICGC, International Cancer Genome Consortium; MBRGs, metastasis and basement membrane**-**related genes; OS, overall survival; PCA, principal component analysis; ROC, receiver operating characteristic; TCGA, The Cancer Genome Atlas; t-SNE, t-distributed stochastic neighbor embedding

Using the median risk score, we categorized the patients into low- and high-risk groups. In TCGA dataset, we observed that the low-risk group had significantly longer survival than the high-risk group (*p* < 0.001) (Fig. 3A). In addition, the receiver operating characteristic (ROC) curve demonstrated that this MBRGs-based prognostic model was highly reliable, with an area under the curve (AUC) of 0.749, 0.714, and 0.706 for 1-, 2-, and 3-year survival, respectively (Fig. 3B). The risk score plot and survival status analysis revealed that the low-risk group exhibited favorable survival outcomes with an extended OS duration. Furthermore, all the risk genes were upregulated in the high-risk subgroup (Fig. 3C). PCA and t-SNE analysis showed that the patients in the two subgroups were further divided into two distribution patterns (Fig. 3D). These findings reveal that the MBRGs prognostic model can be used to accurately forecast the clinical trajectories of patients with HCC.

### Validation of the MBRGs prognostic model in the ICGC database

The LIHC-JP cohort from the ICGC database was used as the validation group to evaluate the universality of the MBRGs prognostic model in the training cohort. The same formula used for TCGA cohort was used to calculate the risk score for each patient in the validation cohorts. The results revealed that patients in the high-risk group had a significantly poorer survival status than those in the low-risk group (*p* = 0.001) (Fig. 3E). Furthermore, ROC analysis demonstrated that the MBRGs prognostic model had AUC values of 0.728, 0.731, and 0.683 for 1-, 2-, and 3-year survival, respectively (Fig. 3F). The distribution plot depicting the risk score, survival status, and expression of the 12 genes indicated a correlation between an increased risk score and higher mortality rates (Fig. 3G). PCA and t-SNE revealed distinct directional distributions between the two risk subgroups (Fig. 3H).

### Analysis of the correlation between the MBRGs prognostic model and clinical features

**Fig. 4:**
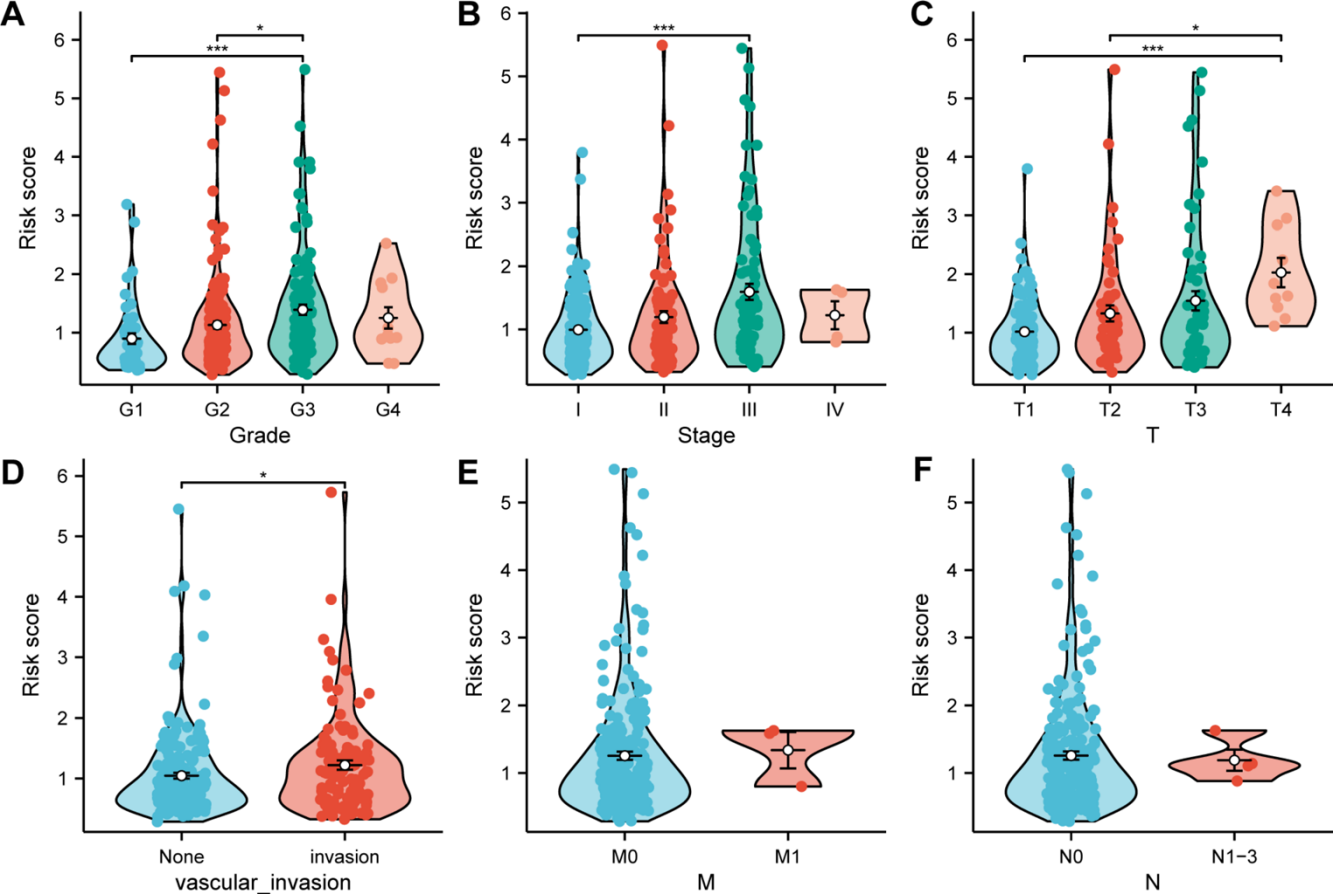
Analysis of the correlation between the MBRGs prognostic model and clinical features. **(A)** Correlation analysis between tumor grade and risk score. **(B)** Correlation analysis between overall cancer stage and risk score. **(C)** Correlation analysis between T stage and risk score. **(D)** Analysis of vascular invasion. **(E)** Correlation analysis between M stage and risk score. **(F)** Correlation analysis between N stage and risk score. *p < 0.05, **p < 0.01, ***p < 0.001. Abbreviations: M, metastasis (stage); MBRGs, metastasis and basement membrane-related genes; N, node (stage); T, tumor (stage)

We conducted further analyses to assess the value of the MRBGs prognostic model in different groups stratified by clinical factors in TCGA cohort. Patients with advanced tumor grade, stage and T stage exhibited higher risk scores (all *p* < 0.05) (Fig. 4A–C). In addition, we found that the high-risk group was more prone to vascular invasion (*p* < 0.05) (Fig. 4D) and observed a trend towards increased risk scores in patients with positive lymph nodes and distant metastasis (Fig. 4E, F).

### Independent prognostic analyses of risk scores and construction of a predictive nomogram model

**Fig. 5:**
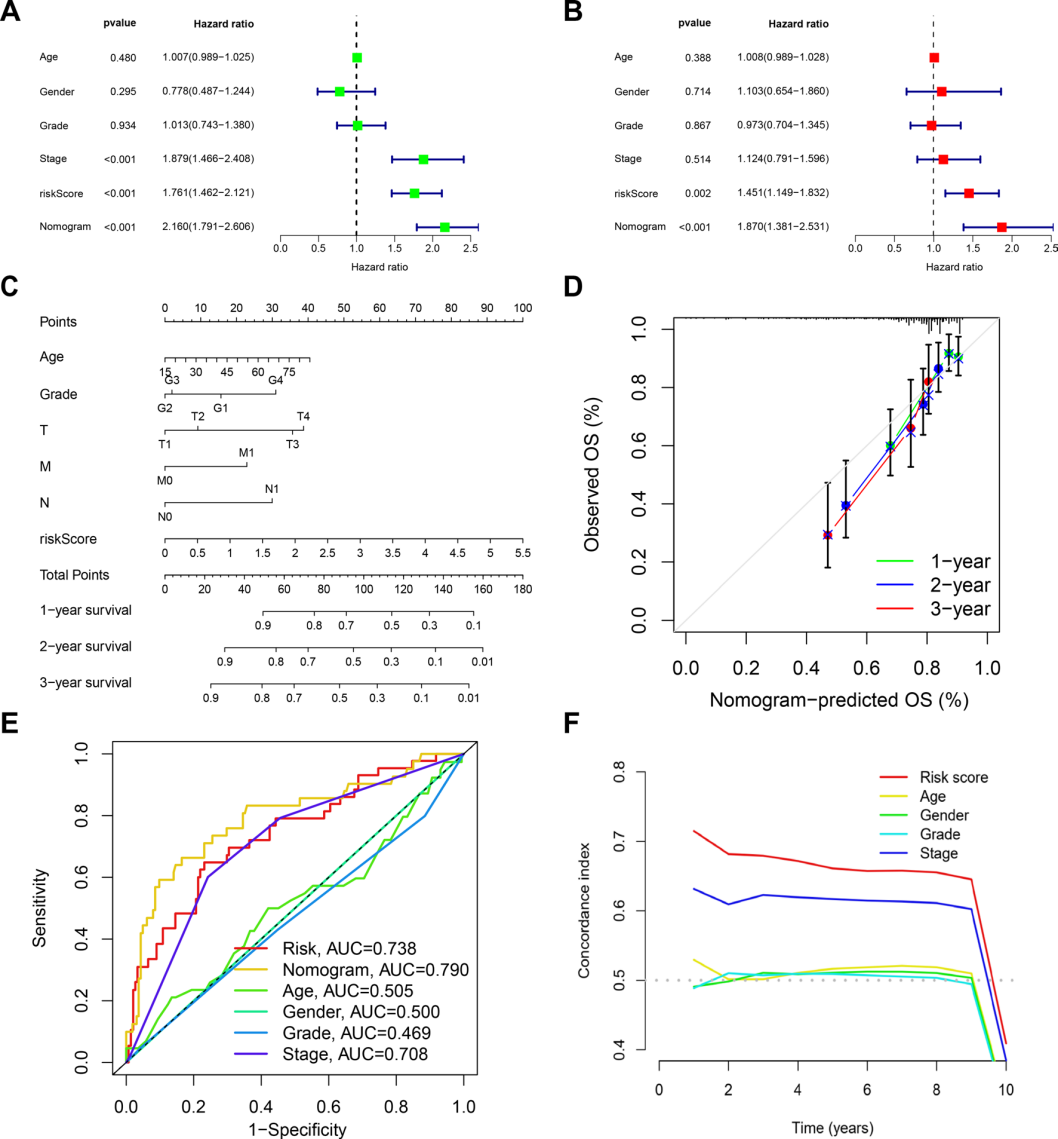
Independent prognostic analyses of risk scores and construction of a predictive nomogram model. **(A)** Univariate analysis of TCGA cohort. **(B)** Multivariate analysis of TCGA cohort. **(C)** Nomogram for predicting survival. **(D)** Calibration curve of the nomogram to assess accuracy. **(E)** Multivariate ROC curve for tumor grade, age, stage, gender, nomogram and risk score. **(F)** Concordance index in the model performance evaluation. Abbreviations: AUC, area under the curve; M, metastasis (stage); N, node (stage); OS, overall survival; ROC, receiver operating characteristic; T, tumor (stage); TCGA, The Cancer Genome Atlas

Univariate and multivariate Cox analyses were used to explore the independent prognostic factors for LIHC. The results showed that the risk score and stage were significantly associated with OS, suggesting that the risk score of the MRBG prognostic model constructed using the TCGA-LIHC cohort was an independent prognostic factor for patients with HCC (*p* < 0.01) (Fig. 5A, B). Therefore, we created a nomogram to assess patient prognosis more accurately based on independent predictive indicators of HCC (Fig. 5C). In addition, the calibration curves (1, 2, and 3 years) demonstrated favorable performance during the internal validation of the nomogram (Fig. 5D). As shown in Fig. 5E, F, the nomogram had a better predictive ability when combined with clinical information, with an AUC of 0.790. Overall, using an MBRGs-based prognostic model for patients with HCC integrated with clinical information, a nomogram with superior credibility and accuracy was successfully constructed.

### MBRGs score of HCC is positively correlated with EMT activity

**Fig. 6:**
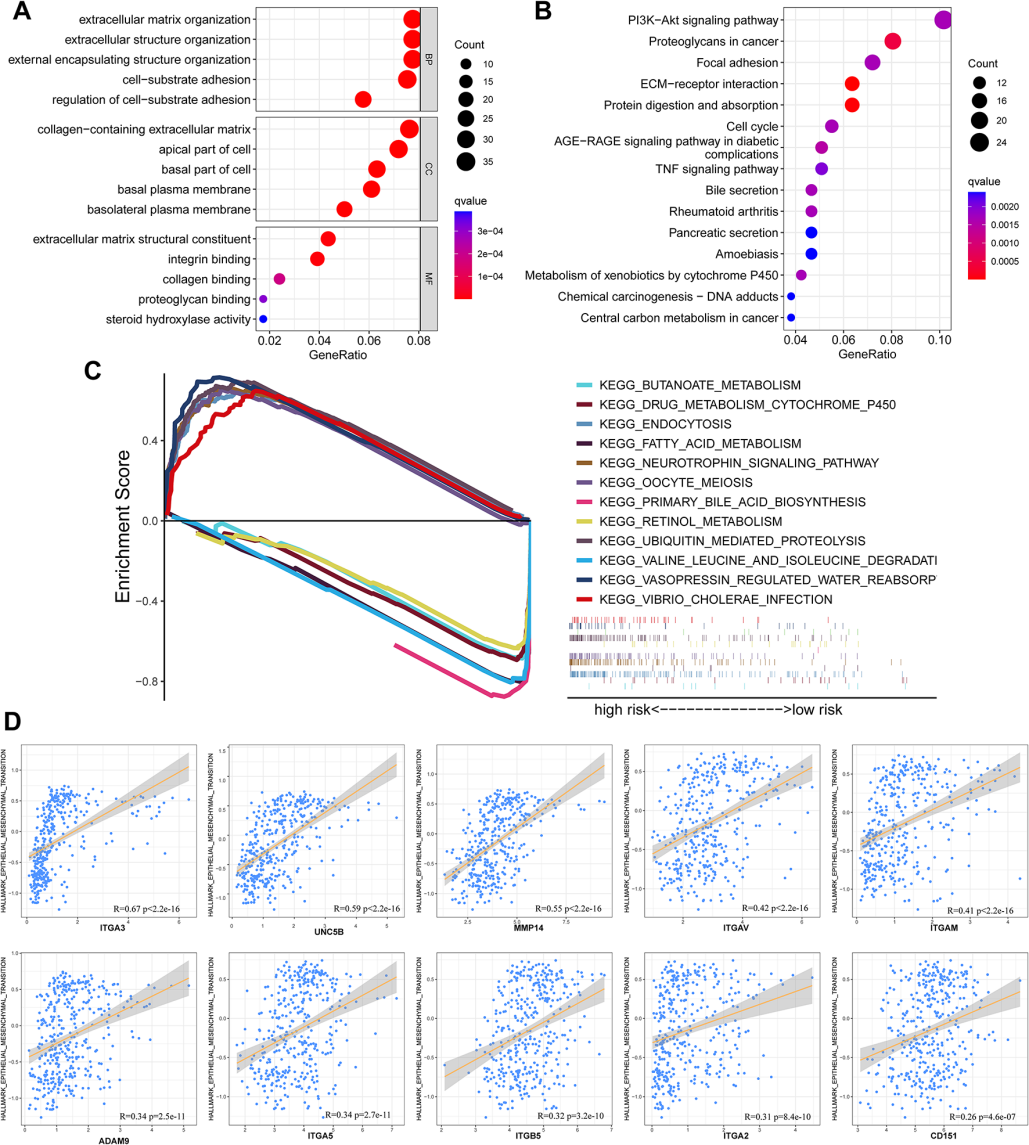
Functional enrichment analyses and correlation of MBRGs expression levels with the EMT pathway. **(A)** GO enrichment pathway analysis plot. **(B)** KEGG enrichment pathway analysis plot. **(C)** GSEA. **(D)** Scatterplots illustrating the expression correlation between the 10 signature genes and the EMT pathway in the hallmark gene set. Abbreviations: EMT, epithelial-mesenchymal transition; GO, Gene Ontology; GSEA, gene set enrichment analysis; KEGG, Kyoto Encyclopedia of Genes and Genomes; MBRGs, metastasis and basement membrane-related genes

Loss of the BM, which occurs during EMT, is believed to be a crucial step in the development of tumor metastasis and malignancy and led us to investigate the correlation between MBRGs score and the EMT pathway. Both KEGG and GO enrichment analyses revealed that the previously identified DEGs were significantly enriched in numerous pathways related to immunity and tumor metastasis (Fig. 6A-B). In the GSEA, the high-risk subgroup was mainly enriched in cell proliferation-related pathways and the low-risk group was mainly enriched in cell metabolism-related pathways (Fig. 6C) (Table S2). These results provide insights into the importance of the BM in HCC progression and metastasis. Notably, GSEA results suggested that the poor prognosis of the high-risk subgroup was mainly due to cell proliferation.

Finally, correlation analysis of scatter plots revealed a strong positive correlation between the EMT pathway and expression of the MBRG *ITGA3* (Fig. 6D).

### Immunological analysis of patients with LIHC in various risk groups

**Fig. 7:**
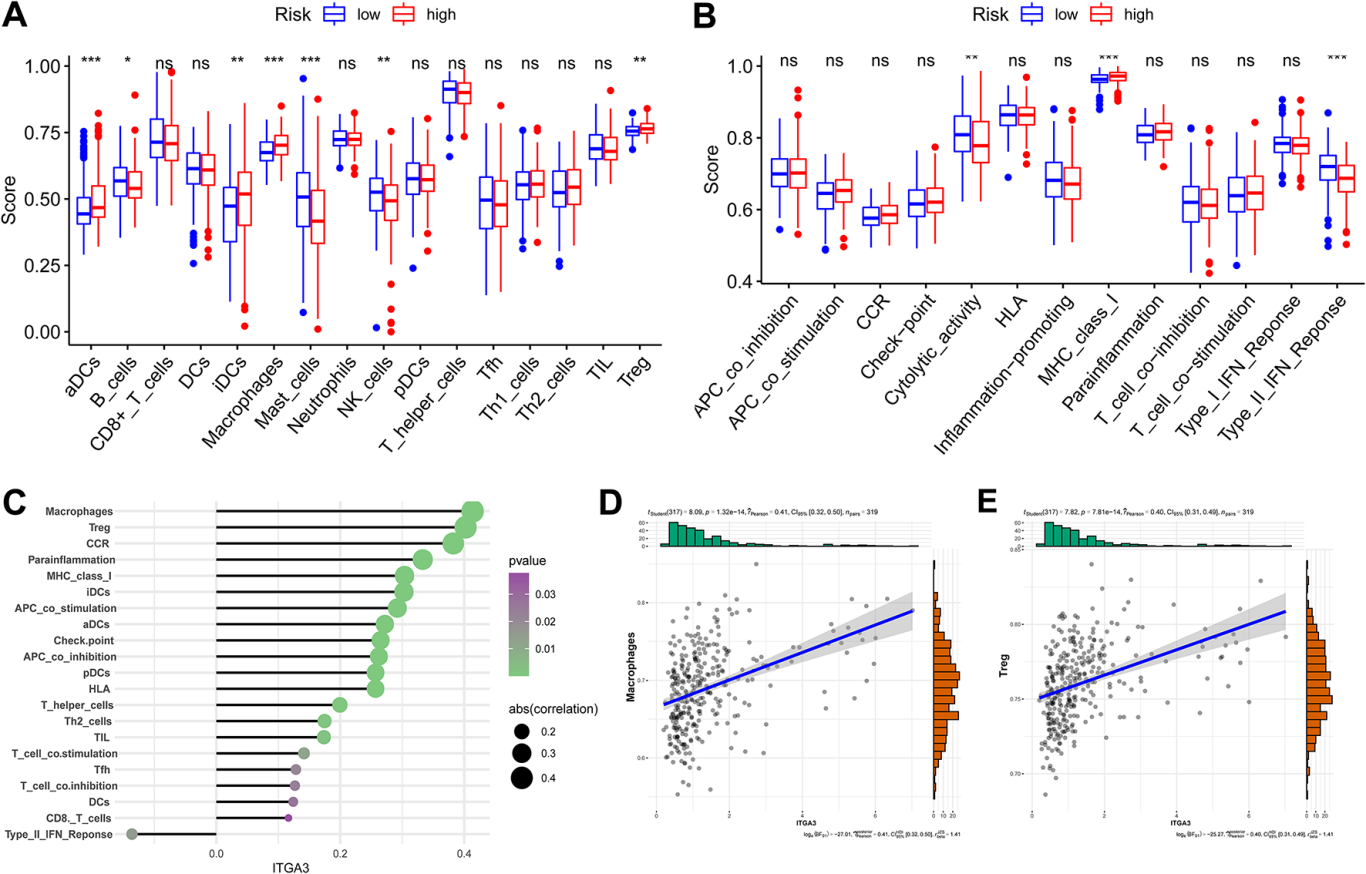
Immunological analysis of LIHC patients in respective risk groups. **(A**,** B)** ssGSEA scores for immune cells and immune function in TCGA cohort. **(C)** Correlation between ITGA3 expression and enrichment of immune cells. **(D)** Correlation between *ITGA3* expression and macrophage enrichment. **(E)** Correlation between *ITGA3* expression and Treg enrichment. ns = *p* > 0.05 (not significant), *p < 0.05, **p < 0.01, ***p < 0.001. Abbreviations: APC, antigen-presenting cell; aDC, activated dendritic cell; CCR, chemokine receptor; DC, dendritic cell; HLA, human leukocyte antigen; iDC, immature dendritic cell; INF, interferon; *ITGA3*, integrin subunit alpha 3; LIHC, liver hepatocellular carcinoma; MHC, major histocompatibility complex; NK, natural killer; pDC, plasmacytoid dendritic cell; ssGSEA, single-sample gene set enrichment analysis; TCGA, The Cancer Genome Atlas; Tfh, T follicular helper; TIL, tumor-infiltrating; Treg, regulatory T cell

To further explore the correlation between risk score and immune status, we evaluated the enrichment scores of 16 immune cell types using ssGSEA in the high- and low-risk groups in TCGA. The results showed significant differences in immune cell infiltration between the two subgroups. For example, activated dendritic cells, immature dendritic cells, macrophages, and Tregs were significantly enriched in the high-risk subgroup, whereas B cells, mast cells, natural killer (NK) cells, and other immune cells were mainly enriched in the low-risk subgroup (*p* < 0.05) (Fig. 7A). Besides, in immune function, MHC_class_I had higher levels in the high-risk group, while Cytolytic_activity and Type_II_IFN_Reponse had higher levels in the low-risk group (*p* < 0.05) (Fig. 7B). In addition, *ITGA3* expression was positively correlated with a variety of immune cells and immune functions, such as macrophages and Tregs (*p* < 0.05) (Fig. 7C–E). These results revealed a similar immune status between the ICGC and TCGA cohorts and suggest that the poor prognosis of patients in the high-risk group could be caused by high tumor-associated macrophages (TAMs) status and Treg levels and low levels of NK cells.

In the TIMER, CIBERSORT-ABS, QUANTISEQ, and MCP counter algorithms, immune cells, particularly T cells, were more abundant in the high-risk group (Fig. S3), indicating that immunotherapy is more beneficial in the high-risk category.

### MBRGs mutations and immunotherapeutic response: TMD, tumor immune dysfunction and exclusion, and immune checkpoint profiling in different risk groups

**Fig. 8:**
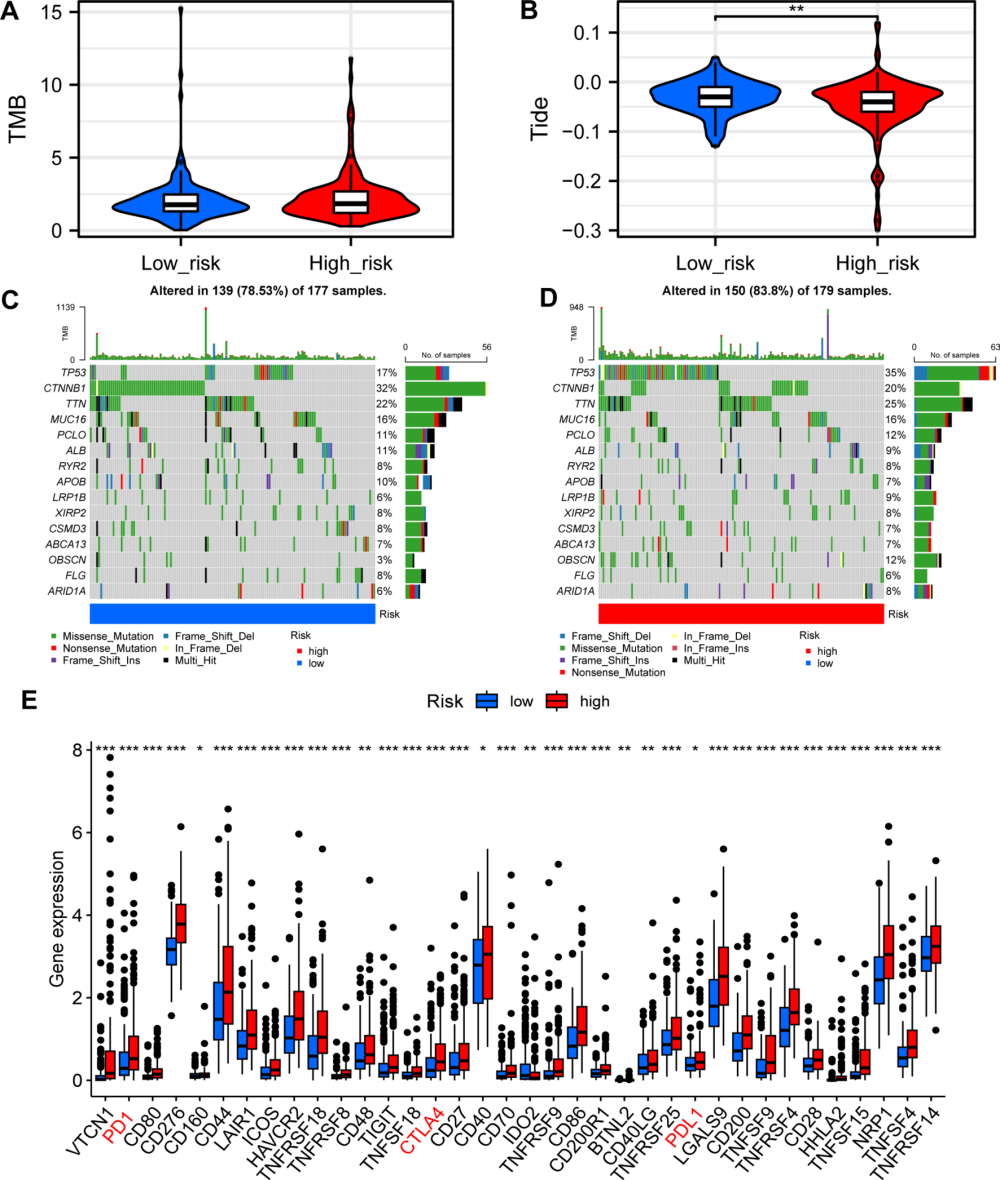
Mutations in MBRGs and immunotherapeutic response. **(A)** TMB analysis. **(B)** TIDE analysis. **(C**,** D)** Differences in mutation incidence between the high- and low-risk groups. **(E)** Differences in the expression of immune checkpoint genes between the high- and low-risk groups. *p < 0.05, **p < 0.01, ***p < 0.001. Abbreviations: DEL, deletion; INS, insertion; TIDE, tumor immune dysfunction and exclusion; TMB, tumor mutation burden

Considering that TMB values, tumor immune dysfunction and exclusion (TIDE) score, mutation frequency, and expression of immune checkpoint genes are closely related to the efficacy of immunotherapy, they were all estimated based on MRBGs in the high- and low-risk groups. TMB quantification analyses demonstrated that the high-risk group had a higher TMB value (Fig. 8A). As shown in Fig. 8B, the TIDE score was lower in the high-risk group, indicating a lower likelihood of immune evasion. Subsequently, we used the “mafTools” R package to further investigate the distribution pattern of the top 15 TCGA-based somatic mutations between the high- and low-risk groups (*p* < 0.01). *TP53* was identified as the most frequently mutated gene in both subgroups, with the high-risk group exhibiting an 18% higher mutation frequency than the low-risk group (Fig. 8C, D). In addition, *TTN*, *LRR1B* and *OBSCN* were significantly upregulated in the high risk group and *CTNNB1* was significantly downregulated in the low-risk group. Overall, the high-risk group had higher mutation frequencies across all factors, indicating that immunotherapy had a greater impact. Moreover, additional investigation of the differences in the expression of immune checkpoint blockage factors between the high- and low-risk groups revealed that *PD-1*, *CTLA4*, and *PD-L1* were highly expressed in the high-risk group (*p* < 0.05) (Fig. 8E). In summary, the high-risk group had a higher TMB value, a higher mutation frequency, increased immune checkpoint gene expression, and a lower TIDE score, suggesting that the high-risk group may have better immunotherapy outcomes.

### Drug sensitivity analysis

**Fig. 9:**
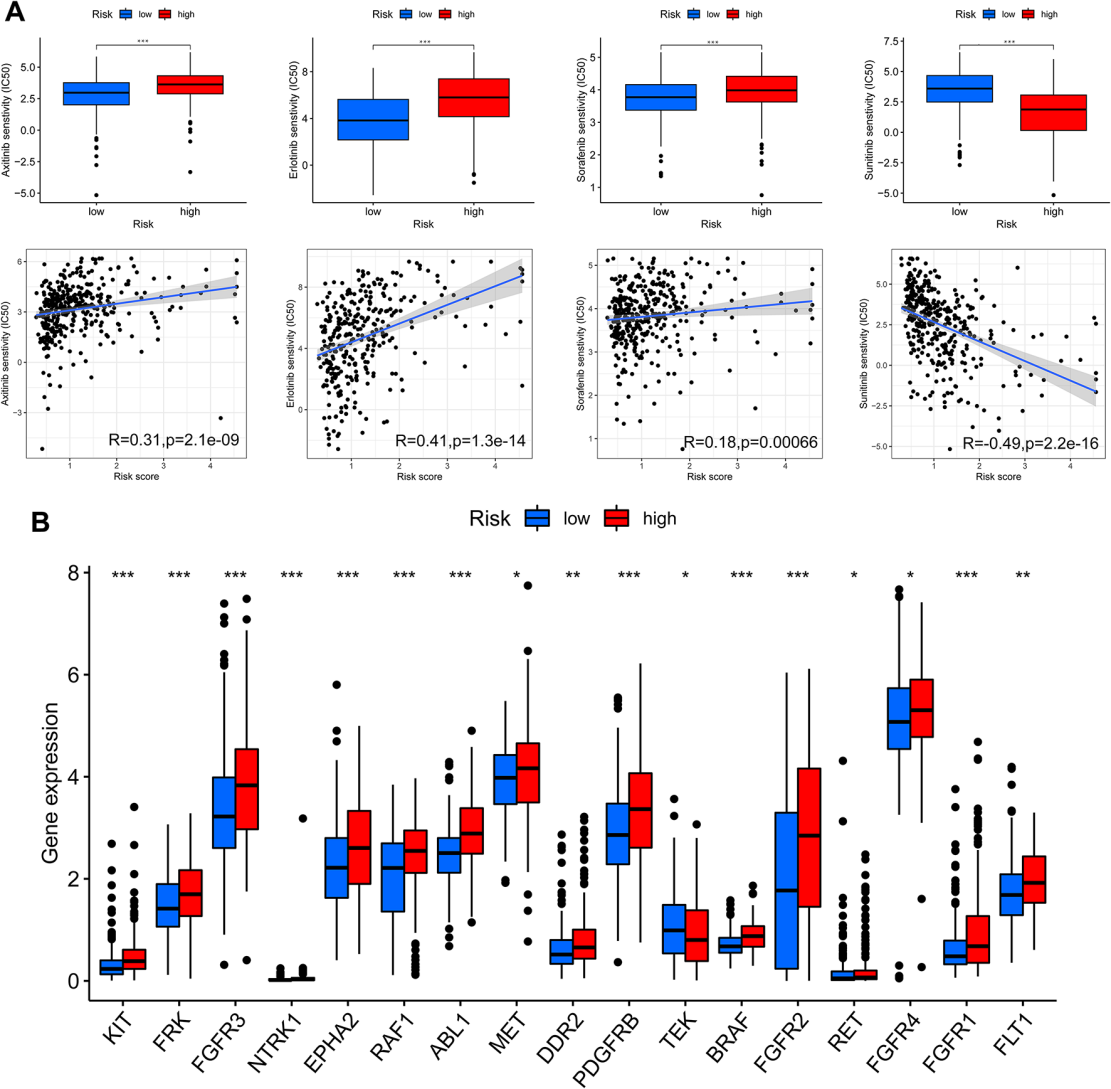
Drug sensitivity analysis. **(A)** Sensitivity analyses of axitinib, erlotinib, sorafenib, and sunitinib in the high- and low-risk patient groups. **(B)** Differences in target gene expression between the low- and high-risk groups. *p < 0.05, **p < 0.01, ***p < 0.001. Abbreviations: IC50, half-maximal inhibitor concentration

To identify potential therapeutic drugs for patients with HCC, we used the pRophetic algorithm to explore the correlation between the MBRG risk scores of patients with HCC and their response to four commonly used anticancer drugs (axitinib, anlotinib, sorafenib, and sunitinib). By calculating the IC50 values for these drugs in both the low- and high-risk groups, patients in the low-risk group were found to exhibit greater sensitivity to axitinib, erlotinib, and sorafenib whereas patients in the high-risk group showed increased sensitivity to sunitinib (Fig. 9A). These trends were supported by the positive correlation between the IC50 values for axitinib, erlotinib, and sorafenib and risk scores and the negative correlation between the IC50 values for sunitinib and risk scores (*p* < 0.001). Furthermore, we obtained target genes from the Drugbank database and assessed their expression in both risk groups based on targeted therapy. Notably, the expression of all target genes was significantly different between the two groups (*p* < 0.05) (Fig. 9B). These findings suggest that risk scores may distinguish patients who are more suitable for appropriate treatment.

### ScRNA-seq analysis and construction of a PPI network

**Fig. 10:**
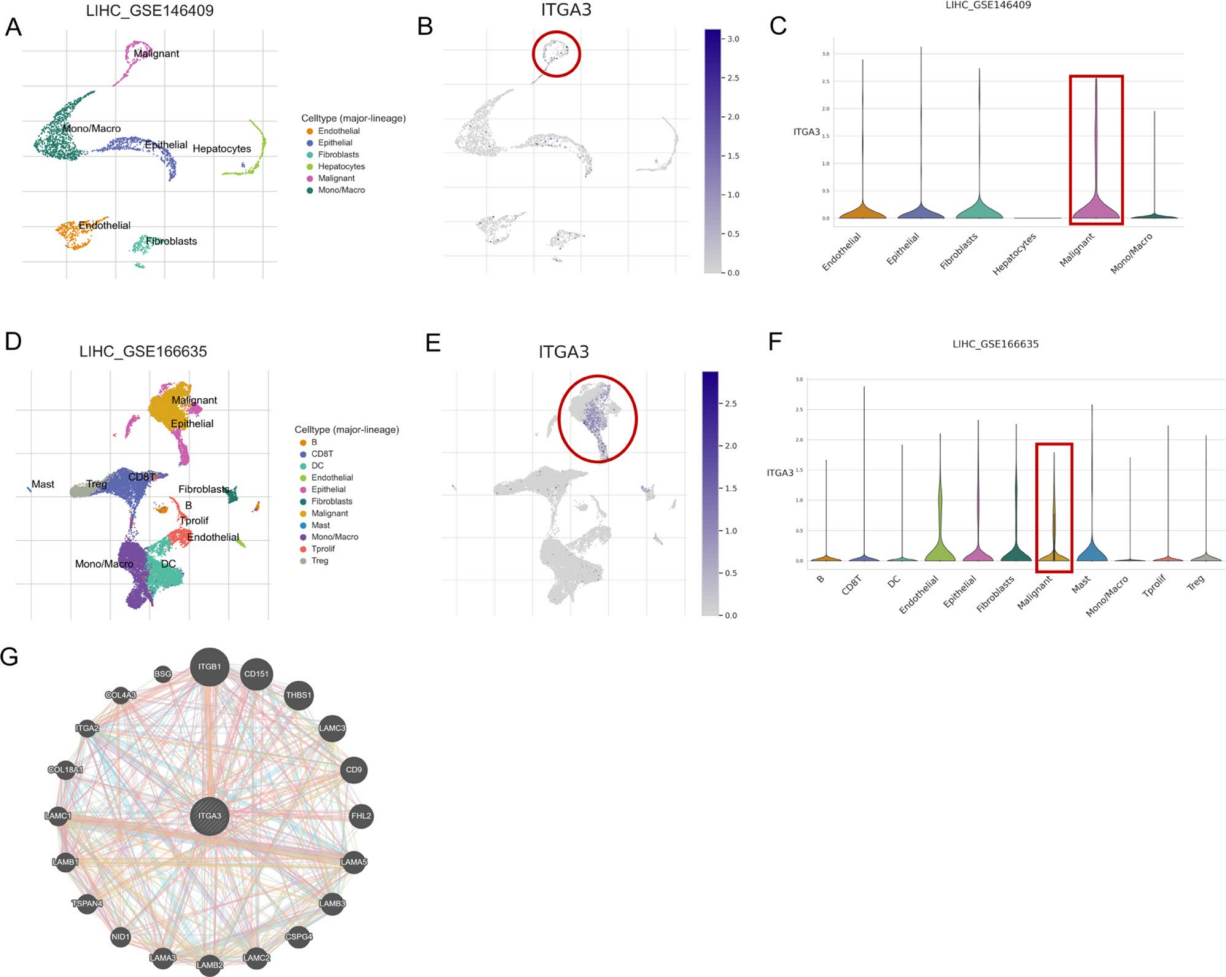
Single cell RNA sequencing analysis and construction of a PPI network. **(A-C)** T-SNE projection of all cells and *ITGA3* expression from LIHC-GSE146409. **(D-F)** T-SNE projection of all cells and *ITGA3* expression from LIHC-GSE166635. **(G)** PPI network centered on *ITGA3*.Abbreviations: *ITGA3*, integrin subunit alpha 3; PPI, protein-protein interaction;

To explore the role of *ITGA3* in HCC at the single-cell level, we performed scRNA-seq analysis using the TISCH 2.0 database. Figure 10A–F illustrates the high expression of *ITGA3* in malignant liver cells. Notably, we found a significantly higher level of *ITGA3* expression in malignant cells compared to hepatocytes in LIHC_GSE146409. We concluded that the high expression of *ITGA3* in HCC cells likely contributes to the poor prognosis of HCC.

To elucidate the co-expression of and interaction between proteins, we selected the highly prognostic gene *ITGA3* for further analysis and built a PPI network using the GeneMAINA database centered on this factor. The resulting PPI network showed that 20 proteins interact with *ITGA3*, with ITGB1 showing the highest correlation with *ITGA3* (Fig. 10G).

### ITGA3 plays a crucial role in the migration and invasion of HCC cells

**Fig. 11:**
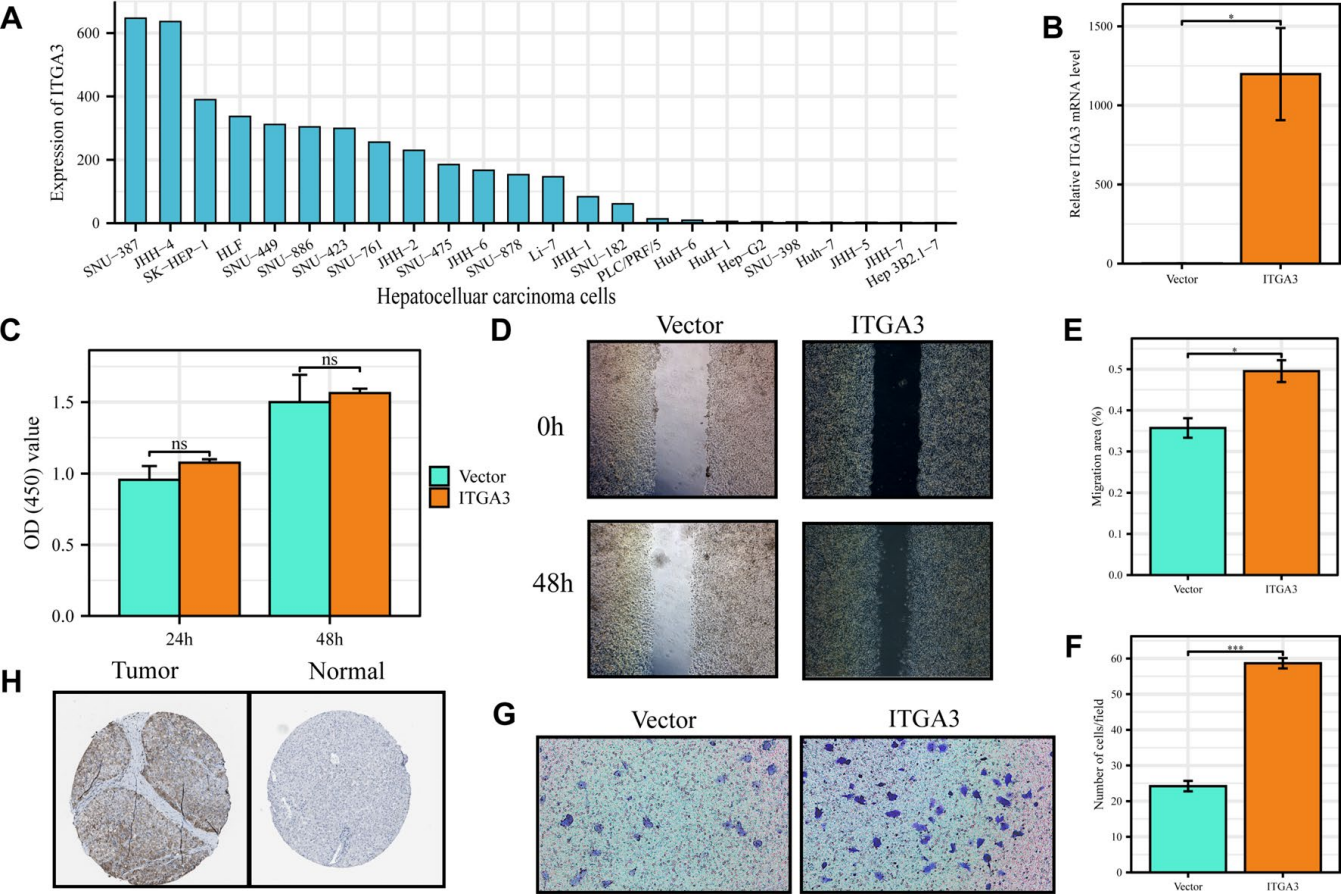
*ITGA3* plays a crucial role in the migration and invasion of HCC cells. **(A)***ITGA3* mRNA expression profile in HCC cells from the HPA database. **(B)***ITGA3* expression after transfection with *ITGA3*-OE lentivirus. **(C)** Results of the CCK8 assay. **(D**,** E)** Results of wound healing assay. **(G**,** F)** Results of the transwell migration assay. **(H)** Representative immunohistochemical results of *ITGA3* expression in HCC and normal liver tissues from the HPA database. *p < 0.05, **p < 0.01, ***p < 0.001. Abbreviations: CCK-8, cell counting kit-8; HCC, hepatocellular carcinoma; HPA, Human Protein Atlas; *ITGA3*, integrin subunit alpha 3; OE, overexpression; RT-qPCR, reverse transcription-quantitative PCR

Our study revealed a strong positive correlation between the MBRGs and EMT pathway, with *ITGA3* being the most strongly correlated MBRG. However, the effect of this gene on the proliferation and migration of HCC has not yet been reported. Because *ITGA3* is expressed at low levels in HepG2 cells (Fig. 11A), an overexpression vector was used to analyze its potential role in HCC. RT-qPCR results showed that *ITGA3* expression was significantly upregulated after transfection with the *ITGA3*-OE lentivirus (Fig. 11B). The CCK-8 assay results suggested that overexpressing *ITGA3* did not affect HepG2 cell proliferation (Fig. 11C). Next, we performed in vitro wound healing and transwell migration assays and found that overexpressing *ITGA3* enhanced the migratory ability of HepG2 cells and significantly increased the number of invasive cells (Fig. 11D-G). In addition, immunohistochemistry results from the HPA database demonstrated that *ITGA3* expression was higher in tumors compared to that in normal tissues (Fig. 11H). These results indicate that *ITGA3* plays a crucial role in the migration and invasion of HCC cells.

## Discussion

In this study, a novel prognostic model for HCC based on MBRGs was developed and served as an effective indicator for predicting the prognosis and response of patients with HCC to immunotherapy. An external dataset containing 240 cases from the ICGC database was used for validation. As expected, the resulting nomogram showed strong reliability, and the ROC curves for each cohort showed the robustness of the MBRGs prognostic model. Furthermore, GO and KEGG analyses and GSEA were performed on patients in the high- and low-risk groups to obtain enrichment pathways. In addition, five immune-related algorithms (CIBERSORT, QUANTISEQ, MCP counter, ssGSEA, and TIMER) were used to examine the immune landscape and activity across the risk groups. The efficacy of immunotherapy in the high- and low-risk groups was assessed by estimating TMB values, TIDE scores, mutation frequencies, and expression of immune checkpoint genes. Subsequently, drug sensitivity analyses were performed on the high- and low-risk groups to predict potential therapeutic agents for patients with HCC. Finally, wound healing and transwell assays were conducted to elucidate the role of *ITGA3* in tumor metastasis.

According to previous studies, all MBRGs play essential roles in tumor etiology. Twelve genes were involved in the proposed model, six of which (*ITGB5*, *ITGAV*, *ITGAM*, *ITGA5*, *ITGA3*, and *ITGA2*) belong to the integrin family. Integrins are cell adhesion and signaling proteins that are essential for a wide range of biological functions and have been strongly implicated in both tumor metastasis- and BM-related functions [47]. In fact, integrin expression is strongly associated with the progression and prognosis of HCC [4], and numerous studies have shown that integrin genes are overexpressed in HCC tissues and mediate HCC cell invasion and metastasis [48, 49]. *MMP14* and *MMP1*, members of the MMP family, can directly cleave almost all extracellular matrix components and participate in the degradation of BM and extracellular matrices, thus promoting tumor invasion and metastasis [50–52]. Overexpression of both *MMP14* and *MMP1* is also closely associated with invasive metastasis and poor prognosis in HCC [50, 51]. According to earlier research, *MMP14* increases the secretion of pre-*MMP-2* and pre-*MMP-9*, degrades the extracellular matrix, and interacts with TIMP-2 to promote metastasis. [50]. Furthermore, *MMP14* can act as a key molecule in the CXCL10/TLR4/MMP14 signaling pathway to mobilize myeloid-derived suppressor cells and promote post-transplant HCC recurrence [53]. The MBRG *ROBO1*, a member of the Ig receptor superfamily, is overexpressed in HCC. It can promote angiogenesis in HCC through the guanosine triphosphatase Rho family and Slit-ROBO signaling pathways and facilitate HCC metastasis by activating the Slit2-ROBO1 signaling pathway [54–56], indicating that the Silt/ROBO signaling pathway may be an effective therapeutic target for HCC. The MBRG *CD151* is a member of the tetraspanin family associated with the promotion of metastasis and plays a pro-metastatic role in various cancers [57]. Several studies have shown that *CD151* upregulation may be a sensitive predictor of HCC metastasis [58] and may induce metastasis in an integrin β1-dependent manner [59]. *CD151* also induces *MMP9* expression and promotes extracellular matrix degradation and cancer cell migration, which contribute to HCC metastasis [60, 61]. Furthermore, *CD151* is positively correlated with the aggressiveness of HCC and is a marker and potential therapeutic target for predicting HCC prognosis [57]. The final MBRG identified in this study was *ADAM9*. Overexpression of *ADAM9*, a zinc metalloproteinase expressed on the cell surface, is thought to be associated with the clinicopathological features of HCC leading to tumorigenesis, invasion, metastasis, and poor prognosis [62]. Although these genes are closely related to hepatocellular pathogenesis, our study is the first to combine them as prognostic markers in patients with HCC.

GO and KEGG functional enrichment analyses showed that altered risk scores were mainly associated with the PI3K-Akt signaling pathway and proteoglycan in cancer, adhesion bands, and extracellular matrix receptor interactions; GSEA analyses showed that the high-risk group was predominantly enriched in cell proliferation-related pathways. Therefore, the poor prognosis of HCC may be related to the PI3K-Akt signaling pathway and cell proliferation. Some studies have shown that platelet-derived growth factor (PDGF) induces MMP expression through PI3K-mediated signaling pathways and that MMP expression may promote EMT by cleaving BM components [63]. Additionally, activation of the PI3K signaling pathway induces EMT, thereby promoting tumor cell endocytosis into peripheral vessels and metastasis to new organs [64, 65]. In addition, PI3K/Akt signaling can be activated upon GPCR stimulation of Ras, thereby regulating cancer cell proliferation and survival [66, 67]. Cell proliferation directly or indirectly influences tumor invasion, and the forces generated during cell division may promote cancer invasion by directly weakening the BM [8]. Therefore, MBRGs may induce the expression of MMPs by activating the PI3K/Akt signaling pathway to degrade the BM and extracellular matrix, promote the occurrence of EMT to disrupt vascular and intercellular connections, or weaken the BM directly, thereby promoting tumor invasion and metastasis.

Over the past few years, immunotherapy has been established as a primary cancer therapy with significant activity and therapeutic potential in a wide range of tumors [68], and a large body of preclinical and clinical studies has highlighted that immunotherapy strategies provide a survival benefit in HCC [69, 70]. Previous studies have reported that TMB values, TIDE scores, expression of immune checkpoint molecules (e.g., PD-L1 and PD-1), and the degree of immune cell infiltration can predict the response of patients with HCC to immunotherapy [71–73]. The results of multiple analyses regarding these factors suggest that the high-risk group may have better immunotherapy outcomes. First, the high-risk group had higher TMB values, possibly indicating that tumor cells could produce new antigens to activate T cells inhibited by immune checkpoints, resulting in a better immunotherapy outcome [74, 75]. Second, the high-risk group had lower TIDE scores, indicating a lower likelihood of immune escape and a greater likelihood that patients would benefit from immunotherapy. Third, most immune checkpoint genes, including PD-L1 and PD-1, the expression levels of which are positively correlated with anti-PD1 or PD-L1 immunotherapy, were highly expressed in the high-risk group [76]. Finally, five algorithms, including ssGSEA, TIMER, CIBERSORT-ABS, QUANTISEQ, and MCP counter, provided collective evidence that immune cells were enriched and exhibited higher infiltration levels of macrophages and T cells in the high-risk group. In conclusion, these findings suggest that the high-risk group may benefit from immunotherapy with improved treatment effectiveness [72]. Therefore, our MBRGs prognostic model may provide valuable insights for patients in the selection of more effective antitumor immunotherapies. However, additional validation is needed to understand the role of our risk score in predicting the response of patients with HCC to immunotherapy. Remarkably, sex was not associated with the prognosis of HCC in our independent prognostic analysis of clinical factors, whereas Chen et al. demonstrated that sex is a vital prognostic factor for HCC [77]. This discrepancy may be due to differences in databases, sample size limitations, or differences in the data analysis methods of our study. Future research should aim to include larger, multi-center cohorts and consider sex as a critical factor in the analysis. Understanding the interaction between sex hormones and immune response could lead to more personalized treatment strategies for HCC patients. Furthermore, sex is one of the key elements in the effectiveness and safety of immunotherapy for a wide range of solid tumors. For example, serum IL-1β, IL-4, IL-6, IL-10, GM-CSF, TNF-α, and sPD-L1 has significant sex-related predictive effects on OS in patients with melanoma or non-small cell lung cancer treated with ICIs [78]. In esophageal cancer, immunotherapy is associated with favorable outcomes in men [79]. Additionally, in a large real-world database, immunotherapy was associated with a significant OS benefit in male patients with HCCs, whereas female participants in phase III trials experienced less OS benefit after ICI treatment for advanced HCC [80].

Finally, we found that the expression of 10 of the 12 MBRGs significantly and positively correlated with the EMT pathway. *ITGA3*, one of the genes with the strongest correlation with the EMT pathway in HCC; and the only MBRG that has not been explored in functional experiments, was selected for further analysis. Previous studies have shown that *ITGA3* regulates EMT in a variety of tumors and has the potential for immunotherapy [81]. *ITGA3* expression negatively regulates stemness and EMT processes in breast cancer cells [82], though it was reported to promote metastasis and EMT plasticity in pancreatic cancer [83]. However, its role in HCC cell migration and metastasis was previously unknown. Our experimental results showed that *ITGA3* overexpression promoted HCC cell migration and invasion. To investigate the role of *ITGA3* at the single-cell sequencing level in HCC, we analyzed ScRNA analysis utilizing two datasets (GSE146409 and GSE166635) from the TISCH 2.0 database. In GSE146409, we found that *ITGA3* was highly expressed in tumor cells, which was the same as our previous results of TCGA-LIHC differential analysis. We concluded that the high expression of *ITGA3* might be an important factor causing the poor prognosis of HCC. Therefore, the role of *ITGA3* in the development and metastasis of HCC warrants further investigation.

In this study, a novel biomarker was constructed by combining two features, metastasis and the BM, which are effective in predicting patient metastasis, prognosis, and immunotherapy efficacy and are expected to lead to the development of new potential drugs. However, this study has several limitations. First, the accuracy of the MBRGs prognostic model in predicting prognosis and immune regulation in patients with HCC remains a crucial clinical question that requires the development of clinical guidelines for its use. Furthermore, the mechanisms linking the MBRGs prognostic model to the therapeutic efficacy of HCC drug treatments have yet to be determined, and further experimental validation on a substantial patient cohort is required.

## Conclusion

In conclusion, we developed a novel prognostic model based on MBRGs that serves as an effective indicator for predicting the prognosis and response of patients with HCC to immunotherapy. Therefore, our findings provide promising insights that should help guide physicians in making more accurate and personalized treatment decisions for patients with HCC.

### Electronic supplementary material

Below is the link to the electronic supplementary material.

Supplementary Material 1: Fig. S1. Correlation network of MBRGs with red lines indicating positive correlations

Supplementary Material 2: Fig. S2. (A-L) Kaplan-Meier curves for each prognostic gene

Supplementary Material 3: Fig. S3. Heatmap for immune responses based on TIMER, CIBERSORT-ABS, QUANTISEQ, and MCP_counter in the high- and low- risk groups

Supplementary Material 4: Table S1. Risk coefficients for each gene

Supplementary Material 5: Table S2. Enrichment pathways

## Data Availability

All the original data in this study are available upon reasonable request from the corresponding author.

## Abbreviations

aDC: Activated dendritic cell
APC: Antigen-presenting cell
AUC: Area under the curve
BM: Basement membrane
CCK: 8-Cell counting kit-8
CCR: Chemokine receptor
CIBERSORT: Cell-type Identification By Estimating Relative Subsets Of RNA transcripts
CNV: Copy number variation
DC: Dendritic cell
DEL: Deletion
DEG: Differentially expressed gene
DMEM: Dulbecco’s Modified Eagle Medium
EMT: Epithelial-mesenchymal transition
FDR: False discovery rate
GO: Gene ontology
GSEA: Gene set enrichment analysis
HCC: Hepatocellular carcinoma
HCMDB: Human Cancer Metastasis Database
HLA: Human leukocyte antigen
HPA: Human Protein Atlas
IC50: Half-maximal inhibitor concentration
ICGC: International Cancer Genome Consortium
ICI: Immune checkpoint inhibitor
iDC: Immature dendritic cell
INF: Interferon
INS: Insertion
ITGA3: Integrin subunit alpha 3
KEGG: Kyoto Encyclopedia of Genes and Genomes
LIHC: Liver hepatocellular carcinoma
M: Metastasis (stage)
MAF: Mutation annotation format
MBRGs: Metastasis and basement membrane-related genes
MCP: Microenvironment Cell Populations
MHC: Major histocompatibility complex
MMP: Matrix metalloprotease
N: Node (stage)
NK: Natural killer
OS: Overall survival
PCA: Principal component analysis
pDC: Plasmacytoid dendritic cell
PPI: Protein-protein interaction
PDGF: Platelet-derived growth factor
QUANTISEQ: Quantifying Tumor Immune Signature Events
ROC: Receiver operating characteristic
RT: qPCR-Reverse transcription-quantitative PCR
scRNA: seq-Single-cell RNA sequencing
ssGSEA: Single-sample gene set enrichment analysis
T: Tumor (stage)
TAMs: Tumor-associated macrophages
TCGA: The Cancer Genome Atlas
Tfh: T follicular helper
TIDE: Tumor immune dysfunction and exclusion
TIL: Tumor-infiltrating
TIMER: Tumor IMmune Estimation Resource
TISCH: Tumor Immune Single-Cell Hub
TMB: Tumor mutation burden
TME: Tumor microenvironment
TNM: Tumor-node-metastasis
Tprolif: Proliferating T (cell)
Treg: Regulatory T cells
t-SNE: t-distributed stochastic neighbor embedding
