# Supplementary figures and images for: Metastasis and basement membrane-related signature enhances hepatocellular carcinoma prognosis and diagnosis by integrating single-cell RNA sequencing analysis and immune microenvironment assessment
Source: J Transl Med. 2024 Jul 31;22:711. doi: 10.1186/s12967-024-05493-0 (PMC11293133; doi:10.1186/s12967-024-05493-0)

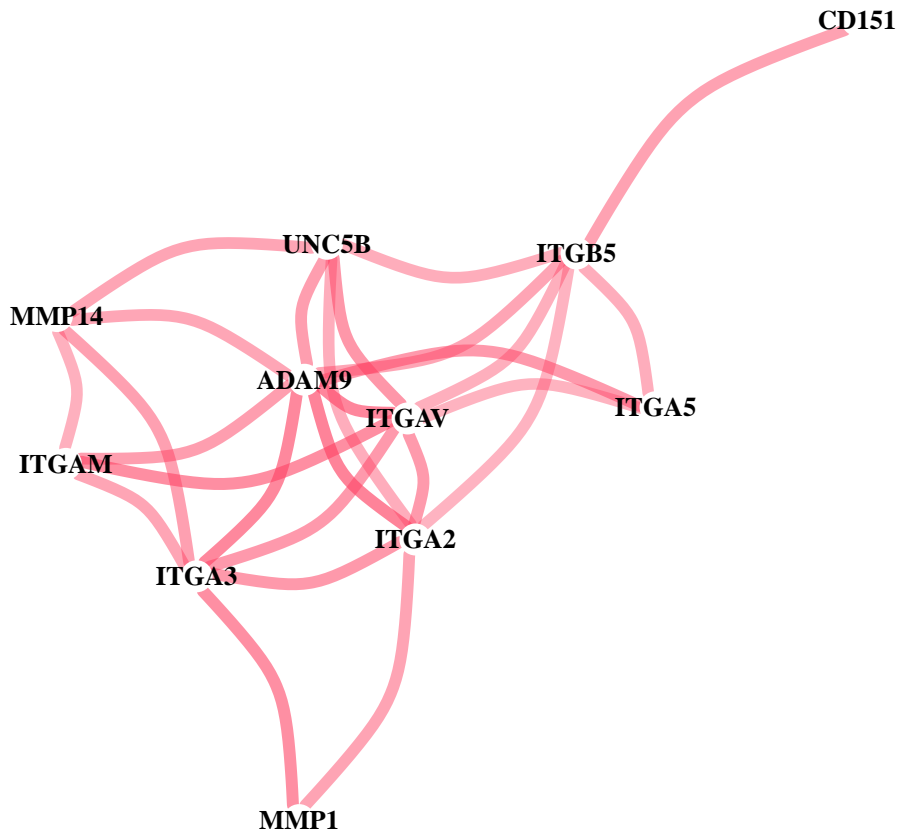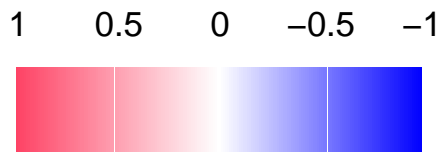

Supplement: Supplementary file 1 — Supplementary Material 1: Fig. S1. Correlation network of MBRGs with red lines indicating positive correlations [file 12967_2024_5493_MOESM1_ESM.pdf]

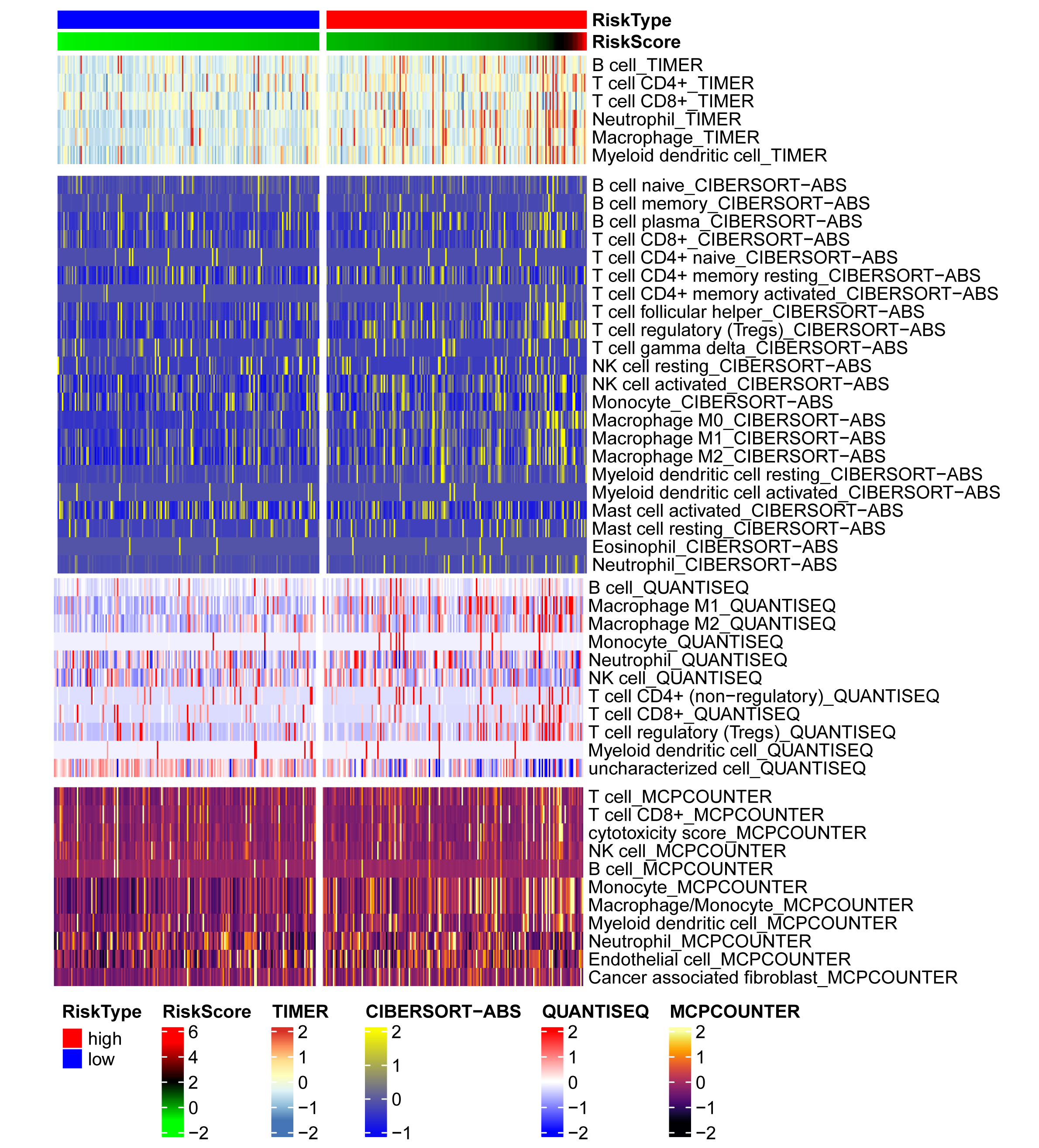

Supplement: Supplementary file 4 — Supplementary Material 4: Table S1. Risk coefficients for each gene [file 12967_2024_5493_MOESM4_ESM.tif]

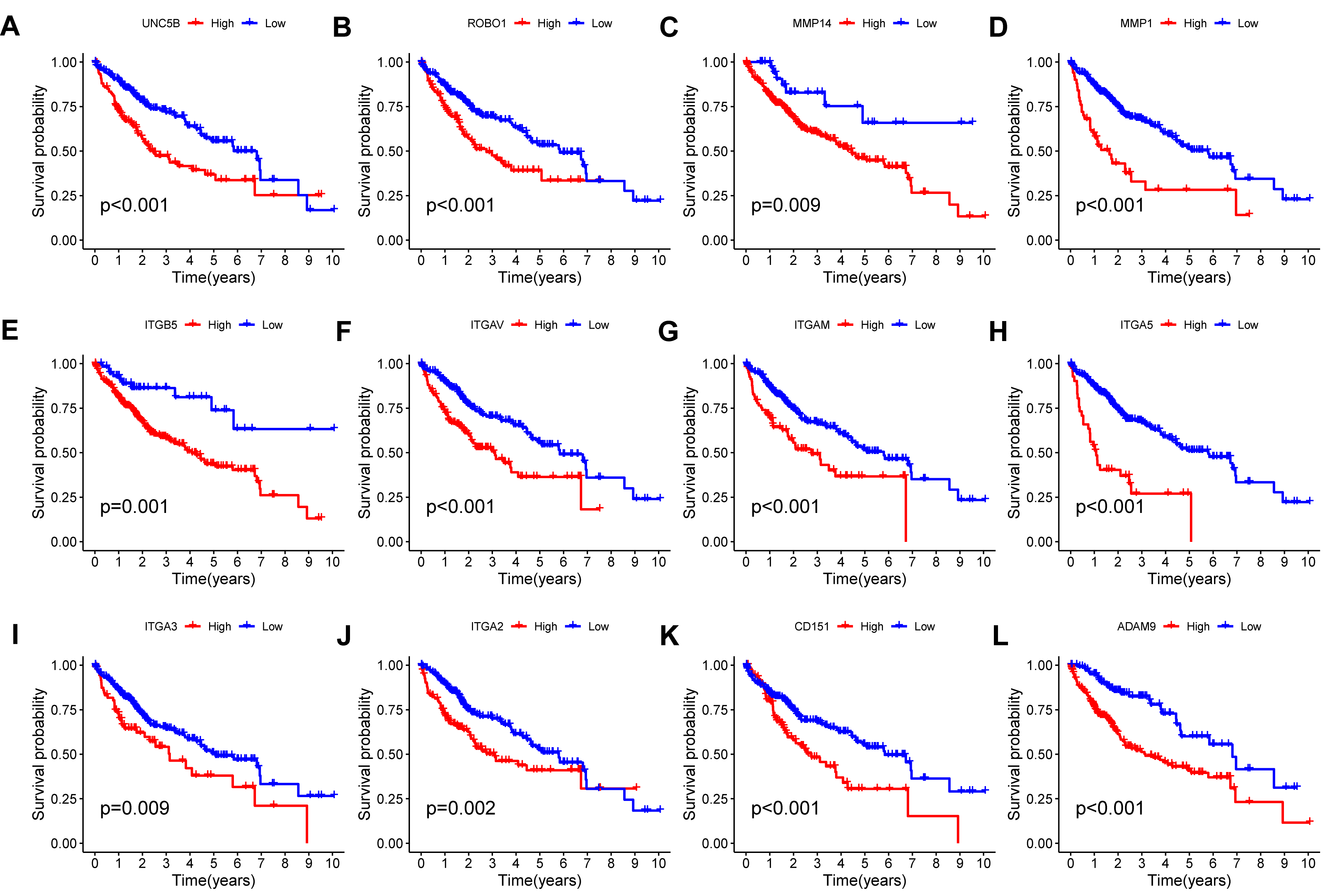

Supplement: Supplementary file 5 — Supplementary Material 5: Table S2. Enrichment pathways [file 12967_2024_5493_MOESM5_ESM.tif]
